# Social Cues and the Online Purchase Intentions of Organic Wine

**DOI:** 10.3390/foods9050643

**Published:** 2020-05-16

**Authors:** Stefanie Sohn, Barbara Seegebarth, Martin Kissling, Tabea Sippel

**Affiliations:** 1 Institute of Marketing, Technische Universität Braunschweig, 38106 Braunschweig, Germany; b.seegebarth@tu-braunschweig.de (B.S.); m.kissling@tu-braunschweig.de (M.K.); t.sippel@tu-braunschweig.de (T.S.); 2 Department of Sociology, Environmental and Business Economics, University of Southern Denmark, 6700 Esbjerg, Denmark

**Keywords:** sustainability, organic wine, store atmospherics, social cues, online retailing

## Abstract

This study investigates how online store atmospherics (i.e., social cues) affect consumer purchase intentions of organic wine. A between-subject experiment with a quantitative survey conducted among German consumers reveals that the mere presence of social cues (i.e., a chat box) on a wine sellers’ online platform positively affects the intention to purchase organic wine from this online store because social cues elicit perceptions of social presence that translate into trust in the online store and brand trust. The latter promotes purchase intentions. Internal (i.e., familiarity with organic wine purchases) and situational (i.e., goal-directedness of shopping) factors do not moderate the effects of social cues.

## 1. Introduction

Against an overall trend of a rather stagnant wine consumption since 2010 [1], online wine sales increased substantially between 2010 and 2017 [2]. Hundreds of online wine sellers including food retailers, specialists and wholesalers are vying for the favor of the customer [2]. In this competitive environment, insights are needed on how to convert visitors of online wine stores to purchasers. Offering organic product alternatives might represent an attractive approach as current forecasts reveal that this wine subcategory will experience the strongest growth among all available wine categories until 2022 [3]. Hence, the overall aim of the current research is to examine the intersection between *organic* and *online* wine purchasing. To provide retailers with information on how to benefit from the current market growth opportunities, this work aims to understand consumers’ purchase intentions of organic wine in online retail environments.

Although scholars have intensively explored the factors determining consumers’ wine preferences [4], the interplay between organic and online wine purchasing has been less well researched. The findings of Quinton and Harridge-March [5] underline the importance to study the online in addition to the offline retail environment to profoundly understand the consumer purchasing of wines. In addition, the findings of Wiedmann et al. [6] show that it is beneficial to distinguish between organic and non-organic alternatives when exploring consumer wine purchasing.

Hence, this study draws on the stimulus-organism-response (SOR) paradigm [7] to elucidate consumer organic wine purchase intentions. Specifically, it examines the role of external factors and particularly, of online store atmospherics (i.e., the bundle of items of information, namely, cues, in an online store environment). Marketing researchers have shown that retail atmospherics might impact product purchasing in offline (e.g., [8]) as well as in online retail settings (e.g., [9]). However, insights are missing on how they affect organic wine purchasing [6]. Their role has also been overlooked with regard to online wine purchasing [10] and wine purchasing in general [4].

To study the effects of online store atmospherics, this research focuses on social cues and examines how the mere presence of social cues in online retail settings affects consumers’ purchase intentions of organic wine. Cues based on human characteristics (e.g., social cues) are of particular importance in online retailing because they compensate for the impersonal nature of those environments [11]. Through social cues, consumers are given opportunities to experience or interact with humans (e.g., peer buyers). However, their mere presence does not imply an interaction. Moreover, social cues are an example of social factors that also include factors such as social desirability, social identities and social norms [12]. Social desirability, social identities and social norms have been found to be one of the most influential factors in terms of effecting green or sustainable consumer behavior [13]. Based on this knowledge, the current study proposes that social cues might be similarly relevant for sustainable purchasing decisions such as organic wine purchasing. In detail, the present research provides answers to the following questions: To what extent and how does the mere presence of social cues affect the consumer online purchase intentions of organic wine? How do internal (i.e., consumer familiarity with organic wine purchases) and situational factors (i.e., goal-directedness of the shopping task) affect the nature of the relationship between social cues and organic wine purchase intentions?

This study combines social presence theory [14] with trust transfer theory [15] to provide an explanation for why social cues affect consumer purchase intentions of organic wine. In doing so, this work contributes to the existing literature on organic wine purchasing. Further, by studying the interplay between supply-side (i.e., retail atmospherics) and individual factors as well as between supply-side and situational factors, the current work broadens the understanding of the effects of supply-side factors in research on consumer (organic) wine purchasing. This is important because systematic research is lacking regarding the conditions under which specific external factors such as the supply-side factors affect consumer purchasing [16,17,18].

The findings of this research show that the mere presence of social cues on an online store’s website affects the consumer purchasing intentions of organic wine through a serial mediation of social presence and trust perceptions at different levels. Neither individual’s familiarity with organic wine purchases nor the goal-directedness of the shopping task affect the postulated effects of social cues.

## 2. Literature Review

With the EU regulation in 2012 concerning organic wine labeling, academic interest on understanding *organic wine purchasing* has been constantly growing (for an overview, see [16]). However, the existing findings are limited to internal (i.e., demographics [19], knowledge [20,21], habit [19], values [6,20]) and product-related supply-side determinants (i.e., prices [19], origin [22,23], taste [24]). The role of atmospherics as another example of supply-side factors has been overlooked in prior research.

Similarly, references to these effects are missing in the research on consumer *wine purchasing* in general. Although scholars have intensively explored the factors determining consumers’ wine preferences (for an overview, see [4]), the role of retail atmospherics has been considered insufficiently. The findings of these studies provide comprehensive evidence for that internal factors such as gender and age [25], lifestyle [26], nationality [27], knowledge [28] or consumer susceptibility to normative influence [29] determine consumers’ wine purchasing. In addition, first empirical insights show that external factors such as product-related supply-side factors (e.g., packaging information [30], claims and certifications [6]) shape the consumer preference towards wines. Moreover, Dobele, Greenacre, and Fry [31] propose that other external factors such as situational elements (e.g., purchase goals) equally affect the purchasing of wine.

Research on consumers’ wine preferences also considered the emergence of online distribution channels and shed light on factors determining *online wine purchasing*. This research stream acknowledges the pivotal role of internal factors such as consumer perceptions of online wine stores [32,33] and social media usage [34] for online wine purchases. Existing research on this perspective also provides first insights into the role of external or supply-side factors. Lynch and Ariely [35], for instance, demonstrated that decreasing the search cost for quality information in wine-selling online stores reduced consumers’ price sensitivity for wines. Cho, Bonn, and Kang [10] further revealed that perceived website quality moderates the effect of wine attributes on risk perceptions and online wine purchasing. However, evidence for the effects of retail atmospherics is also missing in this research stream.

## 3. Conceptual Model and Hypotheses Development

The SOR paradigm provides an orientation on how atmospherics affect consumers’ approach-avoidance behavior. Particularly when information about product attributes (e.g., taste) is unavailable, consumers assess the offering based on easily accessible information like retail atmospherics [36]. By arguing along similar lines, researchers have empirically demonstrated that retail atmospherics affect product purchasing in offline (e.g., [8]) and online (e.g., [9]) retail settings. Therefore, retailers consciously use atmospherics to shape consumer responses. Given the plethora of available retail atmospheric cues, scholars developed cue classifications. In online settings, they rely on visual elements. Wang and Emurian [37], for instance, distinguish between *graphic* (e.g., background color), *structural* (e.g., layout type), *social* (e.g., comments from other visitors) and *content* (e.g., product images) cues.

The research on (virtual) social presence specifies the potential effects of social cues. While perceived social presence generally reflects the feeling of “being with another in a mediated environment” [38], virtual social presence captures the ability of a website to convey human warmth and sociability [39]. In online environments, social presence can be induced in multiple ways (e.g., avatars) [40] and in particular, through social cues [41]. Social cues such as customer reviews on websites are expected to enhance the perceived social presence of a website [42]. Empirical evidence is, however, sparse on this relationship. An exception represents the work of Fang et al. [43] revealing that the option to immediately share information with online communication partners induces a feeling of social presence. Thus, the current research hypothesizes that

**Hypothesis** **1a (H_1a_).** *The mere presence of social cues on an online stores’ website evokes a higher level of perceived social presence than the absence of social cues.*

Overall, social influence theory postulates that social presence (regardless of whether it is actual, implied or imagined) fosters individuals’ responses in a situation [44]. Empirical research reveals that subjective social presence has predominantly favorable consequences [41]. Specifically, the extant information systems research implies that perceived social presence induces trust among people with downstream consequences (e.g., loyalty) (e.g., [14]). Evidence exists, for instance, that the perceived social presence positively affects trust in an online store (e.g., [42]). Socially rich environments are closely related to trust perceptions because in situations of low social richness, it is easier to identify untrustworthy behavior [14].

Trust is generally described as “an expectation held by one party that the word, promise, or statement of another party can be relied on” [45]. Particularly within the electronic (e-)-commerce context, people rely on trust to overcome the lack of interpersonal exchange [14]. Regarding green or organic purchases, trust also occupies a central role because it is the main element on which relationships are based [46]. In their definition of trust, scholars refer to various entities, namely, trust in e-commerce [39], trust in the online store (e.g., [47]) or trust in the brand [48]. This research investigates two distinct trust perceptions to clarify the relationship between virtual social presence and organic food purchasing. First, this paper examines the effect of social presence on the perceived trust in the online store. By defining trust in the online store as “the willingness of a consumer to be vulnerable to the actions of an online store” [47], this research hypothesizes the following:

**Hypothesis** **1b (H_1b_).** *The perceived social presence positively influences the perceived trust in the online store.*

Second, this study examines the relationship between trust in the online store and trust in the organic wine brand. Here, trust in the brand refers to consumers’ willingness to rely on the brand [48]. Trust transfer theory helps to explain how people develop trust to an (unknown) entity like the wine brand [15,49]. Accordingly, trust in the target entity (e.g., wine brand) can be determined by trust perceptions in another entity related to or surrounding the target entity. Proximity or similarity of the individual entities is a prerequisite to enable such a spillover [49]. Hence, evidence exists that the perceived trust in a salesperson, for instance, affects customers’ judgments of brands in a store where the salesperson works [50]. However, empirical evidence is missing on how the perceived trust in the online store affects individuals’ trust in brands of products offered in the online store [49,51], Due to proximity of the entities *wine brand* and *online store* and with reference to the spillover hypothesis, this study proposes:

**Hypothesis** **1c (H_1c_).** *The perceived trust in the online store positively influences the perceived trust in the organic wine brand.*

The extant literature posits that consumer trust reflects a pivotal determinant of consumer purchase intentions [48]. In the context of green product purchasing, brand trust is particularly important, as consumers tend to be skeptical towards green products [52]. Thus, this research hypothesizes as follows:

**Hypothesis** **1d (H_1d_).** *The perceived trust in the organic wine brand positively influences organic wine purchase intentions.*

**Hypothesis** **2 (H_2_).** *The mere presence of social cues affects organic wine purchase intentions via a serial mediation of the perceived social presence, trust in the online store and trust in the organic wine brand.*

The model of consumer response to online shopping suggests that internal factors shape the influence of online stimuli on internal states and approach-avoidance behavior [9]. Accordingly, this study proposes that consumers’ familiarity with organic wine moderates the effect of social cues. Through prior organic wine purchases, consumers get familiar with this product category. Familiarity, in turn, shapes which information people process [53]. People with an enhanced familiarity apply a progress mode in processing. Therefore, they superficially evaluate the displayed products and associated peripheral information such as the social cues on a website. However, consumers with limited familiarity follow a data-driven processing style and apply an assessment mode; they attempt to consider all information carefully [53]. Consistent with this reasoning, this study hypothesizes that consumers who are familiar with purchasing organic wine are likely to minimize the cognitive processing of all surrounding stimuli during purchasing organic wine. This minimization, in turn, prevents them from interpreting the available information cues to form cognitive and behavioral responses [9]. Thus, this study proposes the following hypothesis:

**Hypothesis** **3 (H_3_).** *Consumers’ familiarity with organic wine moderates the relationship between the presence of social cues and organic wine purchase intentions through the perceived social presence, trust in the online store and trust in the organic wine brand. This effect is weaker for consumers who are familiar with organic wine than for consumers who are unfamiliar with organic wine.*

**Figure 1:**
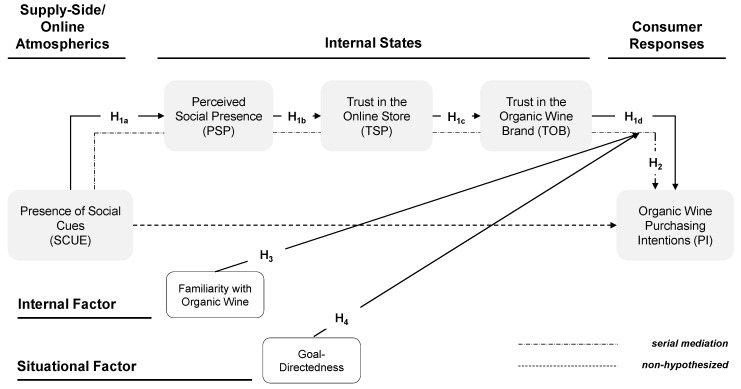
Conceptual model.

The type of shopping task might equally affect how consumer process information on a website because consumers apply task-specific mechanisms while interacting in online environments. That is, they process online stimuli depending on the respective shopping task [54]. Scholars typically classify shopping tasks into the categories of goal-directed or experiential tasks [54]. Goal direction means that consumers seek to complete their purchasing goal, whereas an experiential task describes an undirected browsing behavior. During experiential shopping, consumers use websites in an unstructured manner, preferably for recreation. Experiential users explore websites and put extensive effort into environmental processing, whereas goal-directed users are guided by minimizing effort in processing information cues [55]. Due to the fact that the presence of social cues provides additional information, goal-directedness might result in ignorance or the superficial processing of this information. Thus, the effects of social cues on cognitive and behavioral consumer responses should be weaker during goal-directed than during experiential shopping sessions. Thus, and as demonstrated in our conceptual model (Figure 1), this research hypothesizes as follows:

**Hypothesis** **4 (H_4_).** *The online shopping task moderates the relationship between the presence of social cues and organic wine purchase intentions through the perceived social presence, trust in the online store and trust in the organic wine brand. This effect is weaker for goal directedness than for non-goal directedness.*

## 4. Methods

**Figure 2:**
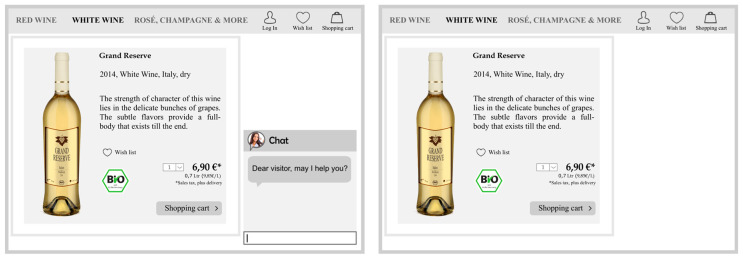
Stimulus material.

This study employs a 2 (presence vs. absence of social cues) × 2 (shopping task: goal directed vs. non-goal directed) between-subject design. For the experiment, two pictures of a fictional online store that sells wine were developed (Figure 2). The pictures created for both the control and the experimental group display a bottle of white wine from a fictitious brand. The displayed website uses conventional website elements (e.g., a shopping basket) and depicts additional product information to enhance external validity. The price was set to 6.90 Euro because this price level represents a favored wine price in Germany [56]. By using the procedure of Pancer, McShane, and Noseworthy, [57], this research inserted the conventional eco-label to present an organic product alternative. To manipulate the presence of social cues, this study follows the procedure applied by Ogonowski et al. [39]. Therefore, the experimental, in contrast with the control group, sees a chat window through which website visitors can interact with the online retailer (Figure 2).

To manipulate the shopping task, the participants were asked to imagine a hypothetical situation. In the goal-directed situation, the participants were told that they were browsing the Internet in search of a wine; in the non-goal directed situation, they were asked to imagine browsing the Internet without any specific goal until an advertisement for the fictional online store appeared and they followed this link. To check the manipulation, a pretest was conducted for which a sample similar to the main study was used (*n* = 70). The manipulation of the shopping task was tested with a scale by Kaltcheva and Weitz [55]. The results of this study indicate that the manipulation worked well (*M_goal_* = 3.92, *M_non-goal_* = 2.10; F(1, 68) = 81.52, *p* < 0.001).

Data collection including the presentation of the experimental stimuli was realized with a standardized online questionnaire in the main study. First, the respondents were asked to indicate their sociodemographic background and their online shopping involvement. Second, the participants were randomly assigned to one of the four scenarios that are a combination of pictures and text. Third, after having contact with one out of the four scenarios, the participants indicated their intention to purchase the depicted product and their trust in the online store and in the organic wine, which were measured on pre-tested 5-point Likert scales (Table 1). Fourth, the participants’ familiarity with the product category (i.e., Have you ever purchased organic wine? [58]) was captured to test H_3_, and the manipulation of the shopping task was tested again with a scale by Kaltcheva and Weitz [59]. Fifth, the participants were shown a set of different screenshots of the online wine store. One of these screenshots was the picture of the online store that the participants actually saw. Accordingly, the participants were asked to select the picture that they thought they had previously seen to ensure the manipulation of the presence of the chat box. Finally, they evaluated the perceived realism of the created online store.

The online survey that contained the experimental websites was distributed via virtual platforms at different German universities. Examining consumer responses in this way guarantees that the participants use their own devices as they typically do when shopping online. The participants were informed that the study explores consumer online shopping behavior. Germany is the largest market for organic wine worldwide [3]. In harmony with previous research (e.g., [62]), a student sample was used to test the hypotheses. Students represent a relatively homogenous respondent population that allows the experimental procedure to be isolated [63]. Additionally, students are appropriate for this type of research due to their frequent Internet use for communication and purchasing [64]. Furthermore, students are current and key future purchasers of organic food and beverages [65] and might thus purchase organic wine either for themselves or as a present.

After removing the participants who provided incomplete answers or who misremembered the online store, the final sample consisted of 647 answers (Table 2). The manipulation of goal-directedness worked as intended (perceived goal-directedness: *M_goal_* = 3.73, *M_non-goal_* = 2.34; F(1,645) = 449.34, *p* < 0.001).

## 5. Results and Discussion

The data were analyzed by using Mplus 7.4 and in particular, with the implemented maximum likelihood procedure with robust standard errors estimator in several steps [66]: (1) this study used confirmatory factor analysis (CFA) to assess the reliability and validity of the employed measures, (2) structural equation modeling (SEM) to test H_1a_–H_1d_, (3) a bootstrapping algorithm to test H_2_, and (4) a multi-group SEM analysis (MGA) to test H_3_ and H_4_.

The results of the CFA indicate acceptable reliability and validity for all measurement models. The factor loadings exceed the recommended threshold of 0.70 and were significant, which signifies indicator reliability and convergent validity [67]. The measures exhibit satisfactory psychometric properties, with an average variance extracted (AVE) of at least 0.612 and a composite reliability (CR) of at least 0.862. All considered constructs discriminate one construct from another. Accordingly, the lowest AVE value exceeds the highest squared inter-construct correlation [68]. Overall, the measurement models fit well with the empirical data because the indicators of model fit met the recommended thresholds. Specifically, the root mean square error of approximation (RMSEA) was less than 0.08, the confirmatory fit index (CFI) and the Tucker Lewis index (TLI) was higher than 0.90 and the standardized root mean square residual (SRMR) was less than 0.08 [69] (Table 1).

**Figure 3:**
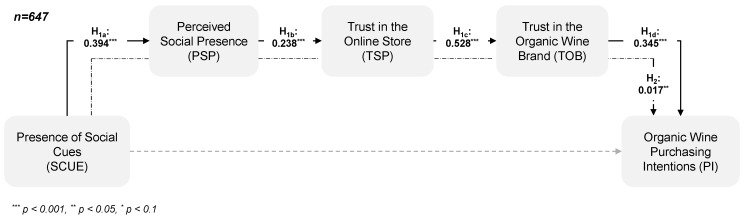
Effects of the presence of social cues on organic wine purchase intentions.

Overall, the structural model was found to adequately fit the data (RMSEA = 0.044, CFI = 0.972, TLI = 0.967, SRMR = 0.064). The results of the data analysis reveal that the mere presence of social cues on an online store’s website indirectly affects the intention to purchase organic wine from this online store through the perceptions of social presence, trust in the online store and trust in the product (H_2_: β = 0.017, CI_95%_ = [0.010, 0.026]). Specifically, the findings demonstrate that the presence of social cues evokes a higher level of perceived social presence than their absence (H_1a_: β = 0.394, *p* < 0.001). Moreover, the perceived social presence positively influences trust in the online store (H_1b_: β = 0.238, *p* < 0.001), which, in turn, increases trust in the brand (H_1c_*:* β = 0.528, *p* < 0.001). Finally, trust in the organic wine brand positively affects purchase intentions (H_1d_: β = 0.345, *p* < 0.001). Accordingly, H_1a–d_ and H_2_ can be accepted (Figure 3).

**Figure 4:**
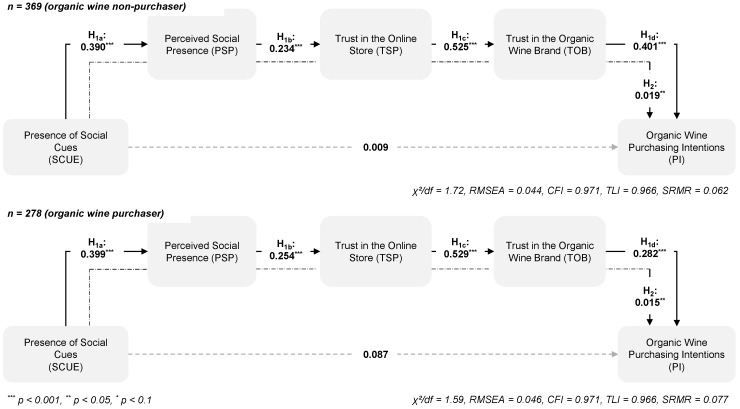
The role of familiarity on the effects of the presence of social cues.

**Figure 5:**
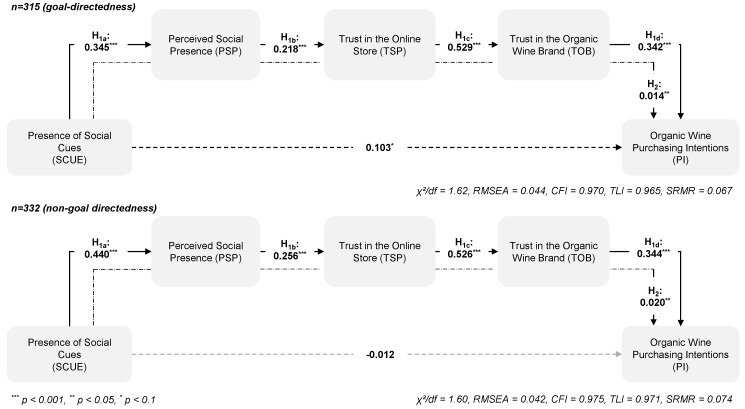
The role of goal-directedness on the effects of the presence of social cues.

To test H_3_ and H_4_, this study examined in a first step whether the measurement models were invariant across samples, namely, *organic wine purchaser* versus *non-purchaser* and *goal-directedness* versus *non-goal directedness*. This study compared a less restrictive model in which all factor loadings were freed across the subsamples with a more restrictive model in which all factor loadings were constrained to be equal across the two subsamples. The χ^2^ difference between the samples of *organic wine purchaser* and *organic wine non-purchaser* (*goal-directedness* versus *non-goal directedness*) was not significant, i.e., Δχ^2^(16) = 12.85, *p* = 0.684 (Δχ^2^(16) = 16.52, *p* = 0.418), which indicates full metric invariance. Thus, the tests for the differences in the indirect effects could be completed to test H_3_ and H_4_. First, this study estimated a structural model with all parameters freed across the respective sub-samples and compared it with a nested model in which the paths were constrained. To test the differences in the indirect effect of social cue presence, a new variable was created in which the difference between the indirect effects was defined. Finally, this study used a bootstrapping procedure (*n* = 10,000) to assess the statistical significance of the differences for the indirect effects. There were neither differences in the indirect effects of the presence of social cues on purchase intentions between *organic wine purchaser* and *organic wine non-purchaser* (H_3_: Δβ = 0.004, CI_95%_ = [−0.026, 0.041], Figure 4) nor between *goal-directedness* versus *non-goal directedness* (H_4_: Δβ = 0.006, CI_95%_ = [−0.026, 0.530], Figure 5). Therefore, hypotheses H_3_ and H_4_ have to be rejected.

As expected, the results show that social cues indirectly affect organic wine purchasing intentions. Existing research on organic wine purchasing supports this finding because it showed that a higher level of information is related to a more positive perception of organic wine [6]. Furthermore, this study’s findings underline the role of supply-side factors to shape consumers’ behavior-related responses towards (organic) wine as identified in previous research [16,70]. However, the current study adds to this perspective by emphasizing the role of wine-unrelated information.

Surprisingly, consumers’ familiarity with organic wine purchasing does not shape the relationships between social cues and purchase intentions, although prior research emphasized the importance of knowledge about organic wine for purchasing decisions [21]. Dahl, Manchanda, and Argo [58], however, found that familiarity with the product purchased does not alter the effects of the imagined social presence; however, it shapes the effect of the physical social presence. In contrast to the initial expectations, the purchase situation and goal directedness during shopping does not shape the effect of social cues on organic wine purchase intentions. The extant research illustrates that consumers distinguish between visual cues or information that are helpful in fulfilling a goal and stimuli that hinder goal completion [71]. Consumers seem to interpret the chat box provided by the retailer as an object that supports their goals.

## 6. Conclusions

This study demonstrates how social cues shape organic wine purchase intentions. In particular, this research reveals that the presence of a chat box on an online stores’ website indirectly influences the consumer purchase intentions of organic wine. More specifically, the presence of a chat box elicits perceptions of social presence that affect trust at different levels; trust perceptions then translate into purchase intentions. Further, it can be stated that both internal (i.e., familiarity with organic wine purchases) and situational factors (i.e., nature of the shopping task) do not influence the effects of the mere presence of a chat box.

This study contributes to different research fields but predominantly enriches the research on the consumer purchasing of organic wine. Overall, this work is the first that sheds light on organic wine purchasing in online settings [16]. More specifically, the present study contributes to the existing research by broadening the understanding on external determinants of consumer organic wine purchasing. Despite the fact, that the extant literature discusses the effects of product-related external factors such as the certification, taste, and packaging [4,16], this study is the first to provide insights into the impact of the product-unrelated retail atmospherics on organic wine purchase intentions. Most importantly, this research uncovers the psychological mechanisms between social cues and organic wine purchase intentions by integrating social presence and trust transfer theory. Furthermore, it demonstrates under which conditions these supply-side factors influence organic wine purchase intentions.

The current study additionally advances the existing knowledge on social factors in the field of sustainable or green consumer research by broadening, on the one hand, the existing scope of social factors [12]. On the other hand, this research uses social presence and trust transfer theory and thus provides an additional explanation for the influential role of social factors on consumer sustainable behavior. Moreover, this study shows that social factors can also exert an important effect on sustainable behaviors in non-public environments such as the online environment [72,73].

This study’s findings also complement the knowledge on the effects of virtual social presence. On the one hand, the present research demonstrates that the mere presence of a chat box elicits perceptions of social presence and thus advance, for instance, existing works on social presence [39,40]. On the other hand, to the best of the authors’ knowledge, this research is the first to examine the impact of social presence on purchase-related outcomes. Prior research focused on the effect of social presence on store-related aspects such as store loyalty [11] and online store trust [14]. Although prior research also referred to purchase intentions as an outcome of perceived social presence [40], they define purchase intentions from a *usage*-related perspective and thus mainly differ from the definition used in this study. The present study adds value by using a *product*-related definition of purchase intentions and by showing that consumers seem to integrate the mere presence of social cues in their virtual shopping environment to form these purchase intentions.

From a practitioner perspective, this study underlines the importance of a conscious selection of atmospheric cues for the design of online stores because they elicit website experiences that guide organic wine purchasing. Retailers can enhance organic wine sales by increasing the level of social cues on their websites, for instance, by using chat boxes. Other social cues that might elicit similar effects are customer reviews or human pictures. Thus, encouraging past purchasers, for instance, to leave a comment and placing these comments close to the organic wine might positively affect future purchasing. Further, practitioners might want to test how these social cues work separately or in combination, also with the aim to create purchase-relevant website experiences. Finally, retailers should try to warrant trust in their online store, for instance, by identifying additional factors that foster website trust perceptions because website trust perceptions confer effects on the purchasing of individual products from the online store. For instance, the presence of website security certificates might evoke the intended effects.

Since these findings rely on selected methods and measures, future research should attempt to broaden the perspective of this study by considering, for instance, additional social cues (e.g., product recommendations) and contrast their effects from the observed effects of chat boxes in this study. One can expect that these options vary in their ability to predict purchase intentions through trust [40]. Moreover, this research studied organic purchase intentions in one product category. Further research should consider different and/or additional product categories. For example, van Doorn and Verhoef [62] showed that consumers’ willingness to pay for organic food depends on the respective product category. Another interesting direction for future research could focus on the long-term effects of social cues. Adaptation level theory suggests that the influence of environmental stimuli on behavior attenuates with repeated exposure [74]. Moreover, the perceived costs resulting from social cues should also attract more attention in future research. Prior research acknowledges, for instance, that atmospheric cues might also raise risk perceptions [75]. Finally, Eastin [76] further postulates that the effect of consumer experience depends on the object of experience. One might assume that this also applies to the effect of consumer familiarity. In contrast to the examined familiarity with the product category, consumers’ familiarity with the online selling platform might shape the effects of social cues, requiring further study. From a methodological perspective, future research should test the documented effects of social cues in a field experiment [46]. Moreover, testing the developed model with another sample might be valuable to overcome the limitations of the current study. The majority of participants in this study had an engineering background. Hence, future sampling should control for gender and/or for study majors.

## Figures and Tables

**Table 1:** Measurement models.

	λ CFA	CR, AVE, *ϕ*^2^
***Perceived Social Presence (PSP)*** [14]		
PSP1: There is a sense of human contact in the website.	0.850	0.903, 0.699, 0.079
PSP2: There is a sense of personalness in the website.	0.858	
PSP3: There is a sense of sociability in the website.	0.797	
PSP4: There is a sense of human warmth in the website.	0.837	
***Trust in Online Store (TOS)*** [47,60]		
TOS1: This store is trustworthy.	0.807	0.888, 0.614, 0.266
TOS2: This store is sincere.	0.793	
TOS3: This store is honest.	0.781	
TOS4: This store is reliable.	0.796	
TOS5: This store wants to be known as one that keeps its promises.	0.740	
***Trust in Organic Wine Brand (TOB)*** [48]		
TOF1: I trust this wine brand.	0.877	0.862, 0.612, 0.266
TOF2: I rely on this wine brand.	0.817	
TOF3: This is an honest wine brand.	0.701	
TOF4: This wine brand is safe.	0.722	
***Organic Wine Purchasing Intention (PI)*** [61]		
OFPI1: I consider buying this wine.	0.840	0.912, 0.777, 0.112
OFPI2: I will go to buy this wine.	0.850	
OFPI3: The likelihood of purchasing this wine is very high.	0.950	
***Model Fit CFA***		
χ^2^/df	1.74	
RMSEA	0.034	
CFI	0.985	
TLI	0.982	
SRMR	0.029	

CFA = confirmatory factor analysis; λ CFA = factor loadings of the CFA; AVE = average variance extracted; *ϕ^2^* highest squared interconstruct correlation; χ^2^ = chi square; df = degrees of freedom; RMSEA = root mean square error of approximation; CFI = confirmatory fit index; TLI = Tucker Lewis index; SRMR = standardized root mean square residual.

**Table 2:** Sociodemographic background of the sample.

***Gender***	
Male	341 (52.7%)
Female	306 (47.3%)
***Academic Subject***	
Arts/Design	3 (0.5%)
Business Administration/Law	27 (4.2%)
Cultural Science	8 (1.2%)
Engineering (Mechanical, Construction, Electrical)	266 (41.1%)
Medicine	11 (1.7%)
Science	85 (13.1%)
Social Science	66 (10.2%)
Teaching	97 (15.0%)
Other	84 (13.0%)
***Desired Degree***	
Bachelor’s	380 (58.7%)
Master’s	226 (34.9%)
Other	41 (6.4%)
***Age***	
M (SD)	23.40 (3.83)
